# Gut microbiota and metabolic health among overweight and obese individuals

**DOI:** 10.1038/s41598-020-76474-8

**Published:** 2020-11-10

**Authors:** Mi-Hyun Kim, Kyung Eun Yun, Jimin Kim, Eunkyo Park, Yoosoo Chang, Seungho Ryu, Hyung-Lae Kim, Han-Na Kim

**Affiliations:** 1 Center for Cohort Studies, Total Healthcare Center, Kangbuk Samsung Hospital, Sungkyunkwan University School of Medicine, Seoul, Republic of Korea; 2 Department of Biochemistry, College of Medicine, Ewha Womans University, Seoul, Republic of Korea; 3 Department of Occupational and Environmental Medicine, Kangbuk Samsung Hospital, Sungkyunkwan University School of Medicine, Seoul, Republic of Korea; 4 Department of Clinical Research Design and Evaluation, SAIHST, Sungkyunkwan University, Seoul, Republic of Korea; 5 Medical Research Institute, Kangbuk Samsung Hospital, Sungkyunkwan University School of Medicine, 29, Saemunan-ro, Jongno-gu, Seoul, 03181 Republic of Korea

**Keywords:** Genetics, Microbiology, Endocrinology, Medical research

## Abstract

Although obesity is associated with numerous diseases, the risks of disease may depend on metabolic health. Associations between the gut microbiota, obesity, and metabolic syndrome have been reported, but differences in microbiomes according to metabolic health in the obese population have not been explored in previous studies. Here, we investigated the composition of gut microbiota according to metabolic health status in obese and overweight subjects. A total of 747 overweight or obese adults were categorized by metabolic health status, and their fecal microbiota were profiled using 16S ribosomal RNA gene sequencing. We classified these adults into a metabolically healthy group (MH, N = 317) without any components of metabolic syndrome or a metabolically unhealthy group (MU, N = 430) defined as having at least one metabolic abnormality. The phylogenetic and non-phylogenetic alpha diversity for gut microbiota were lower in the MU group than the MH group, and there were significant differences in gut microbiota bacterial composition between the two groups. We found that the genus *Oscillospira* and the family Coriobacteriaceae were associated with good metabolic health in the overweight and obese populations. This is the first report to describe gut microbial diversity and composition in metabolically healthy and unhealthy overweight and obese individuals. Modulation of the gut microbiome may help prevent metabolic abnormalities in the obese population.

## Introduction

The prevalence of obesity and obesity-related diseases is growing worldwide. Obesity is a major risk factor for metabolic disease. However, there are obese people who are metabolically healthy, a phenotype called metabolically healthy obesity (MHO). This condition is more often observed in young, physically active patients with a good nutritional status and low levels of ectopic and visceral fat storage. MHO individuals are characterized by a lower degree of systemic inflammation and favorable immune and liver function profiles^1^. Some researchers consider MHO to be a temporary status that eventually develops into metabolic syndrome. Previous studies showed that 33–47.6% of MHO individuals achieved metabolically unhealthy obese (MUO) status over a 5–10 year follow-up period^2^. However, studies have also found that all-cause mortality and risk of metabolic disease and cardiovascular disease (CVD) are lower in the MHO population than the MUO population^3,4^. Furthermore, a recent study revealed that stable MHO subjects were at a lower risk of CVD than MUO subjects, and that metabolic syndrome (MetS) duration was linearly associated with CVD^5^. Many researchers have investigated underlying protective mechanisms in MHO subjects and risk factors for developing metabolic abnormalities, but more studies are needed as the findings from these studies are often conflicting.

Obesity and metabolic disease are complex diseases that result from interaction of genetic and environmental factors. Over the past decade, gut microbiota have been suggested to be an important contributor to the development of obesity and metabolic disease^6^. Gut microbiota play a role through several integration pathways that include the host immune system and response to the environment, including diet^7^. Overall, obesity is associated with gut microbiota that have reduced diversity in terms of composition, which, in turn, reduces metabolic energy consumption in comparison with that of the microbiota of lean individuals^8,9^.

Microbiota have also been shown to be associated with obesity-related comorbidities such as type 2 diabetes (T2D)^10,11^ and metabolic diseases such as hypertension^12^ and dyslipidemia^13^. Modulation of microbiota can reduce metabolic syndrome disorders, suggesting an association between specific microbial composition and metabolic phenotype^14^.

No previous study has evaluated differences in microbiota between metabolically healthy (MH) and metabolically unhealthy (MU) overweight and obese individuals, even though obesity and metabolic syndrome share a range of phenotypes and interactions between genetic risk factors and environmental influences, including the gut microbiota. Debate over the disease risk associated with MHO continues, and there is a lack of understanding of the mechanisms that underlie MHO. For this reason, we evaluated differences in gut microbiota between MH and MU overweight and obese subjects.

## Results

### Subject demographics

Table 1 shows the characteristics of the study population. Among the total of 747 subjects, 317 (42.4%) were in the MH group and 430 (57.6%) in the MU group. MH subjects were more likely to be younger and have a lower body mass index (BMI) than MU subjects. All metabolic indicators except total cholesterol, LDL cholesterol, and hsCRP were significantly higher in the MU group. Age, BMI, and weight were higher in MU group. There was no significant difference in percentage of current smoking between the MH and MU groups. There was no significant difference in nutritional intake between the MH and MU groups (Supplementary Table S1).

**Table 1:** Baseline characteristics of study participants according to metabolic health.

Characteristics	Metabolically healthy (MH)	Metabolically unhealthy (MU)	*p* value^a^
Number (male/female)	317 (249/68)	430 (343/87)	
Age (years)	44.12 (8.20)	47.85 (9.01)	< 0.001
BMI (kg/m^2^)	25.10 (1.62)	26.27 (2.43)	< 0.001
Weight (kg)	72.30 (8.44)	75.25 (10.09)	0.021
Waist circumference (cm)	86.11 (5.73)	89.76 (6.73)	< 0.001
Fat percentage (%)	25.71 (5.90)	27.66 (6.24)	< 0.001
Current smoker (%)	20.2	22.3	0.420
Glucose (mg/dl)	90.90 (5.07)	104.54 (21.81)	< 0.001
Insulin (pmol/l)	5.48 (2.76)	7.66 (4.59)	< 0.001
Systolic BP (mmHg)	109.80 (9.69)	117.51 (13.98)	< 0.001
Diastolic BP (mmHg)	70.62 (7.67)	76.31 (10.87)	< 0.001
HOMA-IR	1.24 (0.65)	2.02 (1.40)	< 0.001
Total cholesterol (mg/dl)	202.51 (32.48)	198.83 (36.56)	0.154
LDL-C (mg/dl)	125.28 (29.20)	122.94(32.60)	0.313
HDL-C (mg/dl)	56.07 (11.26)	49.23 (12.38)	< 0.001
Triglycerides (mg/dl)	119.42 (68.67)	157.02 (88.49)	< 0.001
ALT (U/l)	21.24 (11.90)	29.03 (20.07)	< 0.001
AST (U/l)	21.21 (6.69)	24.26 (10.23)	< 0.001
hsCRP (mg/l)	0.11 (0.25)	0.10 (0.11)	0.546

^a^*p *value for difference between MH and MU groups by *t* test for continuous variables.

### Overall structure of fecal bacterial communities between MU and MH groups: Alpha and beta diversity

**Figure 1:**
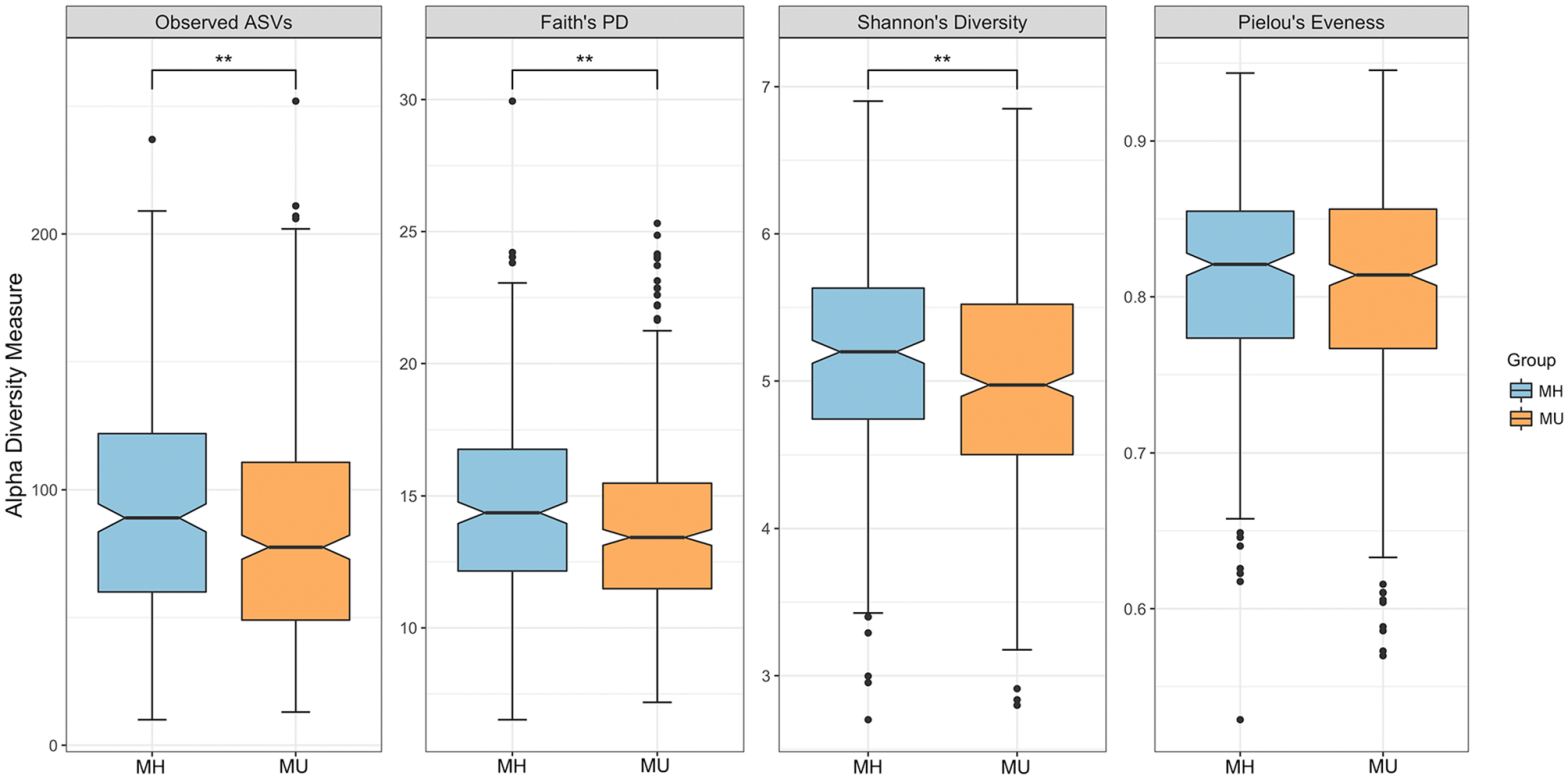
Alpha diversity among groups. Diversity was significant for observed features (ASVs) (*p* = 3.63 × 10^–3^, Kruskal–Wallis test), phylogenetic diversity (*p* = 1.96 × 10^–4^, Kruskal–Wallis test), Shannon index (*p* = 1.03 × 10^–3^, Kruskal–Wallis test), and Pielou’s evenness (*p* = 0.512, Kruskal–Wallis test). *p < 0.05, **p < 0.01. Notched boxes indicate interquartile range (IQR) of 25th to 75th percentiles. The median value is shown as a line within the box, and the notch indicates the 95% confidence interval for the median. Whiskers extend to the most extreme value within 1.5 × IQR. Possible outliers are shown as dots.

The sequencing depth ranged from 2019 to 91,530 reads per sample (mean = 23,970), and the number of features was 2761 in 747 subjects after contingency-based filtering of features. After rarefying the feature tables to 2019 sequences per sample (Supplementary Fig. S2), we found significantly lower richness in both non-phylogenetic **and phylogenetic alpha diversity indices, including observed ASVs (*p* = 3.63 × 10^–3^, Kruskal–Wallis test), Faith’s PD (*p* = 1.96 × 10^–4^, Kruskal–Wallis test), and Shannon’s index (*p* = 1.03 × 10^–3^, Kruskal–Wallis test) in the MU group compared to the MH group; the only exception was Pielou’s evenness (*p* = 0.51, Kruskal–Wallis test) (Fig. 1).

**Figure 2:**
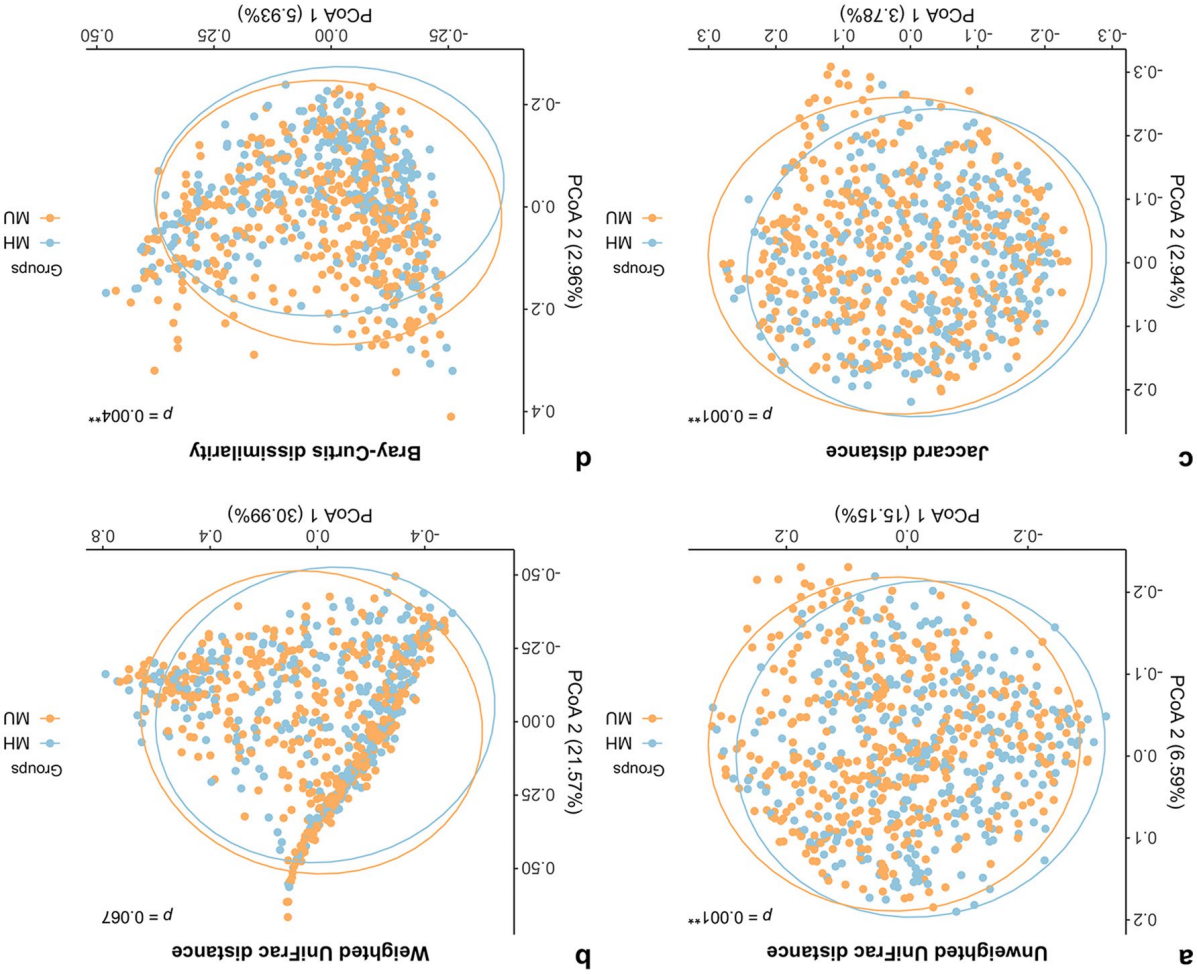
Principal Coordinate Analysis (PCoA) plots of beta diversity. Statistical significance between metabolically healthy (MH) and metabolically unhealthy (MU) groups using distance matrices for beta-diversity: (**a**) unweighted UniFrac distance, (**b**) weighted UniFrac distance, (**c**) Jaccard distance, and (**d**) Bray–Curtis dissimilarity indices. Statistics were calculated using pairwise PERMANOVA with 999 permutations. **p < 0.01. Ellipses represent 95% confidence interval for each group.

Beta diversity analysis indicates the extent of similarities and differences among microbial communities. To quantify beta diversity, both non-phylogenetic (Bray–Curtis dissimilarity, Jaccard distance) and phylogenic methods (Unifrac distance) were used (Fig. 2). We found significant differences between the MH and MU groups in Bray–Curtis (*Pseudo-F* = 1.603, *p* = 0.004, PERMANOVA) and Jaccard (*Pseudo-F* = 1.635, *p* = 0.001, PERMANOVA) non-phylogenetic distances and when using the unweighted UniFrac distance as a phylogenetic index (*Pseudo-F* = 3.815, *p* = 0.001, PERMANOVA). However, due to the large sample number and interindividual variation, fecal microbiota for the MH and MU groups could not be clearly separated by principal coordinates analysis (Fig. 2), even though there were significant differences in microbial community composition between the two groups for all beta diversity indices except weighted UniFrac distance.

In addition, we compared MH and MU microbial diversity with metabolically healthy non-obese (MHN) individuals as the control group. We found significant differences in alpha diversity between the healthy control (MHN) and metabolically unhealthy (MU) groups. The MU group showed lower alpha diversity than both the MHN and MH groups, but there was no difference between the MHN and MH groups (Supplementary Table S2). On the other hand, the microbial structure was different between the MHN and MH, or between the MHN and MU groups in both non-phylogenetic and phylogenetic indices of beta diversity (Supplementary Table S3).

Significant sex differences in gut microbiota were found for alpha diversity. Females showed higher microbial diversity than males in Faith's PD and Shannon's index (Supplementary Fig. S3). We compared the microbial compositions between MH and MU separately for males and females. Sex-separated analyses also showed significantly lower alpha diversity in the MU group than the MH group (Supplementary Table S4), which is consistent with the results of the sex-combined analysis (Fig. 1). For beta diversity, we found the results for males were similar to those of the sex-combined group, but we observed significant differences between the MH and MU groups for only unweighted UniFrac distance in females (Supplementary Table S5). Sex was included as a covariate in the regression model.

### Association of gut microbiota in metabolically healthy overweight/obese subjects and metabolically unhealthy overweight/obese subjects

**Figure 3:**
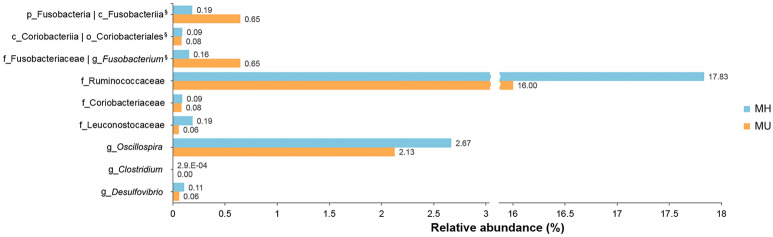
Bar plots for relative abundance of the significant taxa in metabolically healthy (MH) and metabolically unhealthy (MU) groups. The x-axis shows the means proportion of the significantly different taxa between the two groups. ^§^The taxa showed the same relative abundance at both taxa levels.

We investigated the association between gut microbial composition and metabolic healthy status in overweight/obese subjects. To control for covariates, we constructed two models using generalized linear modeling (Table 2). First, we analyzed crude associations without adjustment in Model 1. In Model 2, we controlled for age, sex, and BMI to determine if there was a significant association between microbial taxa and metabolic healthy status in overweight/obese subjects. In Model 1, MaAsLin showed significantly different abundances between the MH and MU groups in three phyla, three classes, three orders, seven families, and five genera. The genus Fusobacterium, including upper level taxa such as Fusobacteria, was more abundant in the MU group than in the MH group (*q* < 0.05). The genus *Oscillospira* in the family Ruminococcaceae showed a strongly negative association with metabolic unhealthy status (coefficient =  − 0.024, *q* = 0.011). We also found that the MU group had a significantly lower abundance of the families Odoribacteraceae, Christensenellaceae, and Coriobacteriaceae than the MH group. Most taxa that were significant in Model 1 remained significant after adjusting for the covariates of age, sex, and BMI (Fig. 3). However, the phylum Actinobacteria, the class Actinobacteria, and their sub-taxa including other Bifidobacteriales and the family Bifidobacteriaceae were not significantly different between the MU and MH groups after this adjustment. Actinobacteria and their sub-taxa were strongly associated with age and sex except taxa in the class Coriobacteriia (Supplementary Table S6). The association of genera *Odoribacter* and *Butyricimonas* with metabolic status was also not statistically significant after adjustment for covariates, although the family Odoribacteraceae still showed significance in Model 2.

**Table 2:** Detection of differentially abundant taxa among groups according to metabolic status.

Taxa	Model 1 (unadjusted)			Model 2 (adjusted for age, sex, and BMI)		
	Coef.^a^	*p value*	*q value*	Coef. ^a^	*p value*	*q value*
**p__Fusobacteria**	0.014	6.09.E−03	0.035*	0.014	7.36. E−03	0.029*
p__Actinobacteria	− 0.013	9.86.E−03	0.035*	− 0.006	2.99.E−01	0.419
p__Tenericutes	− 0.005	1.93.E−02	0.045*	− 0.004	4.82.E−02	0.150
**p__Fusobacteria; c__Fusobacteriia**	0.014	6.09.E−03	0.040*	0.014	7.36.E−03	0.039*
p__Actinobacteria; c__Actinobacteria	− 0.013	7.86.E−03	0.040*	− 0.005	3.04.E−01	0.471
**p__Actinobacteria; c__Coriobacteriia**	− 0.003	1.00.E−02	0.040*	− 0.003	6.24.E−03	0.037*
**p__Fusobacteria; c__Fusobacteriia; o__Fusobacteriales**	0.014	6.09.E−03	0.047*	0.014	7.36.E−03	0.041*
p__Actinobacteria; c__Actinobacteria; o__Bifidobacteriales	− 0.012	9.09.E−03	0.047*	− 0.005	3.27.E−01	0.495
**p__Actinobacteria; c__Coriobacteriia; o__Coriobacteriales**	− 0.003	1.00.E−02	0.047*	− 0.003	6.24.E−03	0.039*
**p__Bacteroidetes; c__Bacteroidia; o__Bacteroidales; f__Odoribacteraceae**	− 0.010	1.71.E−04	0.006**	− 0.007	1.05.E−02	0.045*
**p__Fusobacteria; c__Fusobacteriia; o__Fusobacteriales; f__Fusobacteriaceae**	0.015	6.12.E−04	0.020*	0.015	3.25.E−03	0.020*
**p__Firmicutes; c__Clostridia; o__Clostridiales; f__Ruminococcaceae**	− 0.032	1.64.E−03	0.032*	− 0.032	7.42.E−03	0.034*
p__Firmicutes; c__Clostridia; o__Clostridiales; f__Christensenellaceae	− 0.003	2.36.E−03	0.041*	− 0.002	9.88.E−02	0.217
p__Actinobacteria; c__Actinobacteria; o__Bifidobacteriales; f__Bifidobacteriaceae	− 0.012	4.91.E−03	0.041*	− 0.005	3.27.E−01	0.479
**p__Actinobacteria; c__Coriobacteriia; o__Coriobacteriales; f__Coriobacteriaceae**	− 0.003	8.59.E−03	0.041*	− 0.003	6.24.E−03	0.031*
**p__Firmicutes; c__Bacilli; o__Lactobacillales; f__Leuconostocaceae**	− 0.005	9.09.E−03	0.123	− 0.006	1.17.E−02	0.048*
**p__Firmicutes; c__Clostridia_o__Clostridiales; f__Ruminococcaceae; g__***Oscillospira*	− 0.024	1.00.E−02	0.011*	− 0.022	1.31.E−03	0.015*
p__Bacteroidetes; c__Bacteroidia; o__Bacteroidales; f__Odoribacteraceae; g__*Odoribacter*	− 0.008	5.38.E−02	0.012*	− 0.005	4.82.E−03	0.158
**p__Firmicutes; c__Clostridia; o__Clostridiales; f__Clostridiaceae; g__***Clostridium*	− 0.012	1.89.E−04	0.017*	− 0.012	2.48.E−03	0.021*
**p__Fusobacteria; c__Fusobacteriia; o__Fusobacteriales; f__Fusobacteriaceae; g__***Fusobacterium*	0.013	3.63.E−04	0.028*	0.014	3.41.E−03	0.024*
**p__Proteobacteria; c__Deltaproteobacteria; o__Desulfovibrionales; f__Desulfovibrionaceae; g__Desulfovibrio**	− 0.006	6.12.E−04	0.028*	− 0.006	2.15.E−03	0.020*
p__Bacteroidetes; c__Bacteroidia; o__Bacteroidales; f__Odoribacteraceae; g__*Butyricimonas*	− 0.005	7.66.E−04	0.041*	− 0.004	2.64.E−02	0.102

^a^Coefficients from the generalized linear model using MaAsLin on pairwise testing between two groups. *q < 0.05, **q < 0.01. *q values* were calculated using FDR correction. In Model 2, significant taxa and their *q values* taxa are bolded. p_ = phylum; c_ = class; o_ = order; f_ = family; g_ = genus.

**Figure 4:**
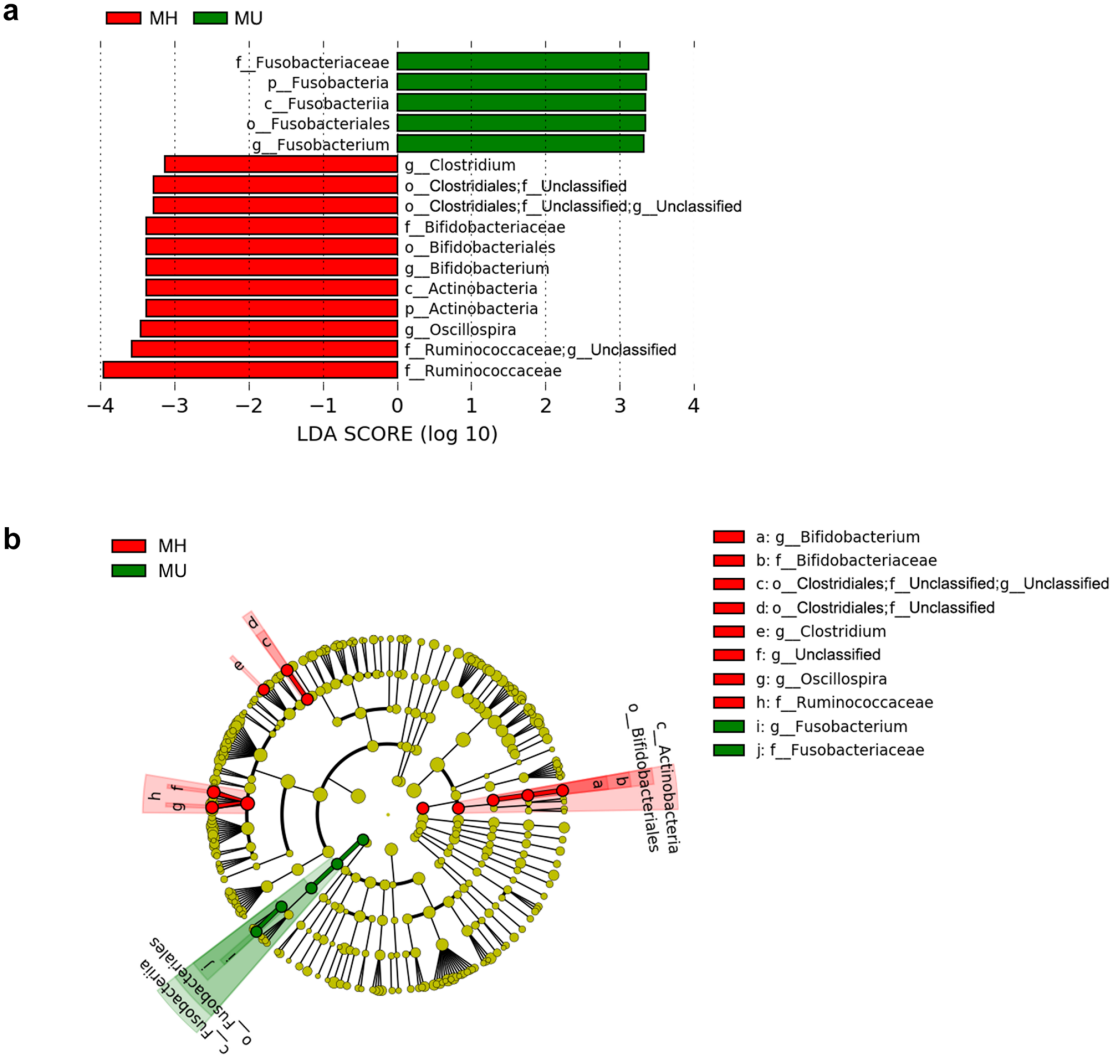
Differentially abundant bacterial taxa in fecal samples from the MH and MU groups in overweight and obese subjects. (**a**) A forest plot showing taxa that were significantly differentially abundant between the MH (red) and MU (green) groups as determined using the Kruskal–Wallis test. LDA score (effect size) indicating significant differences in bacterial taxa (LDA score > 3.0; alpha value *p* < 0.05). (**b**) Cladogram generated using the LEfSe method showing the phylogenetic distribution of microbes associated with the MH and MU groups. Taxonomic levels of phylum, class, and order are labelled, while family and genus are abbreviated. Plots were depicted using LEfSe of Galaxy of the Huttenhower lab.

We also used linear discriminant analysis (LDA) of effect size (LEfSe) to determine the taxa that most likely explained the differences between the MH and MU groups. When performing the LEfSe analysis, we compared taxa not only on the basis of statistical significance, but also based on the biological consistency of the results and effect relevance. Figure 4 shows the LEfSe results (LDA score > 3 and *p* < 0.05) from phylum to genus level, which confirmed that the phylum Fusobacteria and lower taxa including genus *Fusobacteriaceae* were significantly enriched in the MU group. The genera *Clostridium* and *Oscillospira* and the family Ruminococcaceae were enriched in the MH group, consistent with the MaAsLin results. The class Actinobacteria and associated sub-taxa, including Bifidobacteriales, Bifidobacteriaceae, and Bifidobacterium, showed enrichment with a high LDA score in the MH group (Fig. 4), as in Model 1 (Table 2).

### Functional profiling of metabolically heathy/unhealthy obese status

**Figure 5:**
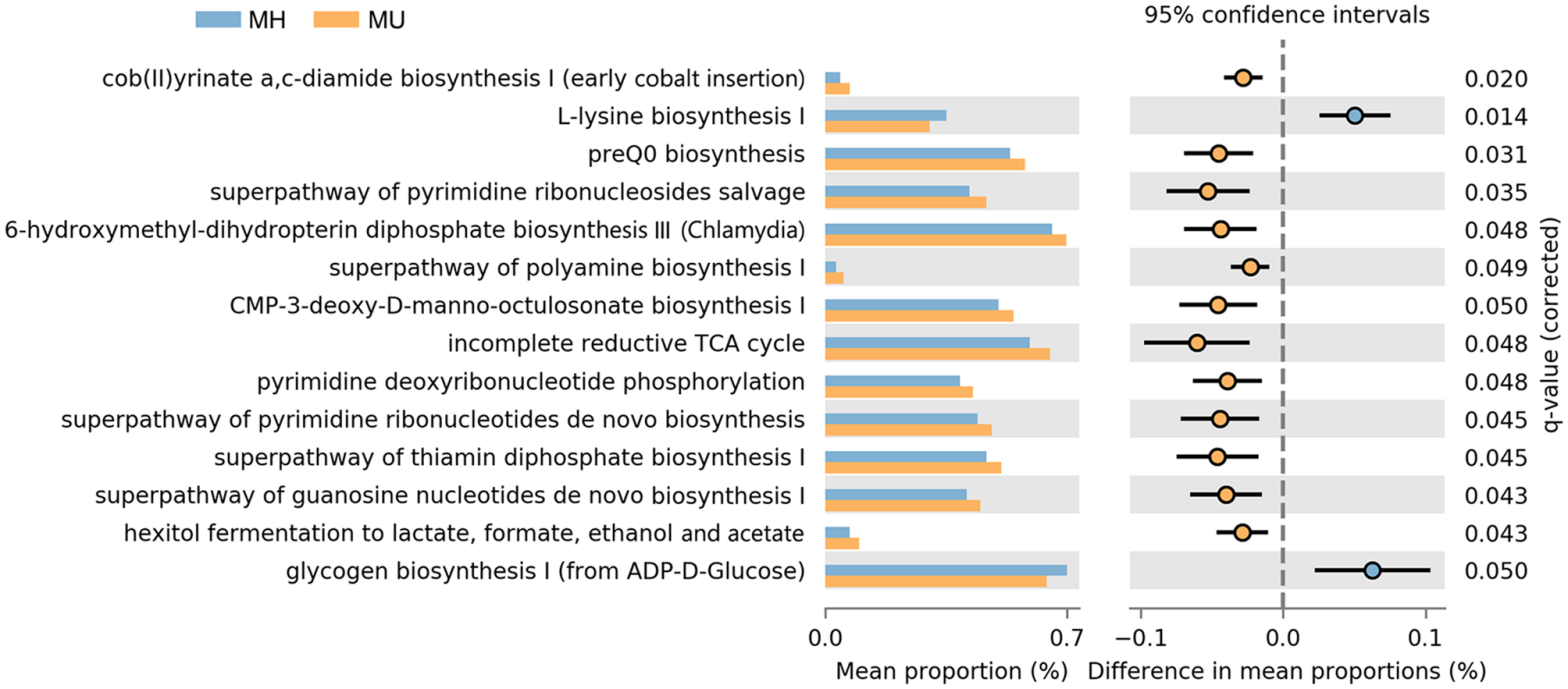
Prediction of metagenome functional content correlated with MH and MU groups using PICRUSt2. Extended error bar plot for each pathway indicating differences in mean proportions for each pair of groups. Two-tailed Welch’s *t* test produced a *q* < 0.05, which was adjusted using the Benjamini–Hochberg method (FDR).

To evaluate differences in community functional attributes, we used PICRUSt2. Among the predicted MetaCyc pathways inferred by PICRUSt2 for ASVs, we found 14 with statistically significant differences between the two groups of subjects (FDR *q* < 0.05) (Fig. 5). The vitamin biosynthesis-related pathways of cob(II)yrinate a,c-diamide biosynthesis I, preQ0 biosynthesis, 6-hydroxymethyl-dihydropterin diphosphate biosynthesis III, and the superpathway of thiamin diphosphate biosynthesis I were enriched in the MU group compared to the MH group (*q* < 0.01). The l-lysine biosynthesis pathway was decreased in the MU group compared to the MH group. Glycogen biosynthesis I was also decreased in the MU group. Nucleotide biosynthesis pathways, such as pyrimidine and purine pathways, were enriched in the MU group.

## Discussion

In this study, we found that metabolic health was associated with gut microbiota composition and abundance in the obese/overweight population. Phylogenetic and non-phylogenetic measures of alpha diversity for gut microbiota were lower in the MU group than the MH group, and there were significant differences in the composition of gut microbiota between the two groups in this study. In previous studies, microbiome diversity and community compositions have been shown to be associated with metabolic components. Decreased gut microbial richness and altered compositions were observed in hypertensive individuals compared to normotensive controls^12^, and gut microbiome diversity was negatively correlated with triglyceride (TG) level and positively correlated with high density lipoprotein cholesterol (HDL-C) level^13^.

Consistent with these previous studies, we found differences in gut microbiota according to metabolic status in overweight and obese individuals. At the genus level, *Oscillospira* within the family Ruminococcaceae and *Clostridium* within the family Clostridiaceae were significantly more abundant in metabolically healthy subjects. Some *Oscillospira* species can likely secrete important short chain fatty acids (SCFAs)^15^ which are a source of energy for the host and can produce a signal through membrane receptors to integrate metabolic functions^1^. SCFAs have beneficial effects on body weight control, inflammatory status, and insulin sensitivity, as well as glucose and lipid homeostasis. Animal studies suggest that SCFAs and succinate have important roles in the prevention and treatment of obesity-associated insulin resistance^16^. *Clostridium* is a butyrate-producing bacterium. Previous studies showed a significant decrease in butyrate-producing bacteria, including *Clostridium*, in individuals with type 2 diabetes mellitus (T2DM) compared to healthy individuals. These results suggest that butyrate-producing bacteria afford protection against T2DM^17^. Butyrate improves colon mucosal barrier function. Moreover, butyrate exhibits immunomodulatory effects and exhibits anti-inflammatory properties by downregulating pro-inflammatory cytokines^18^.

Notably, the family Coriobacteriaceae of Actinobacteria and Leuconostocaceae of Firmicutes were more abundant in the MH group than in the MU group. The family Coriobacteriaceae is known to perform important metabolic functions such as conversion of bile acid, steroids, and phytoestrogens, and this family has been investigated in the context of metabolic diseases^19^. Bacteria have also been reported to play important roles in the onset and maintenance of fatty liver disease^20,21^, although our knowledge of the underlying molecular mechanisms is limited. With the exception of the species *Eggerthella lenta*, one or more members of the family Coriobacteriaceae are considered potential contributors to various biological host functions such as glucose homeostasis and bile acid and lipid metabolism^19,22^, suggesting that the increased presence of Coriobacteriaceae in the MH group may be beneficial. The family Leuconostocaceae comprise lactic acid bacteria (LAB) belonging to the order Lactobacillales; this family was abundant in the MH group. In our previous study, we found a negative association between the family Leuconostocaceae and obese individuals with NAFLD but not lean individuals with NAFLD or all individuals with NAFLD^23^.

We also observed an increased abundance of Fusobacteria in the MU group, including lower taxa levels, supporting a previous study that showed higher abundance of *Fusobacterium* in the gut microbiota of T2DM subjects than in controls, with no significant correlation between BMI and *Fusobacterium*^24^. A majority of studies reported that the Fusobacteria and sub-taxa were enriched in intestinal inflammation^25^. Our results suggest that inflammation-related bacteria such as Fusobacteria might affect metabolic health status in obese and overweight individuals. Except for the Fusobacteria, we found no association with the other phyla. The changes in Firmicutes/Bacteroidetes (F/B) ratio have been reported in obese patients^26^ and in T2DM^10^. In this study, there was no significant difference in the F/B ratio between the MH and MU groups (Supplementary Fig. S4). In spite of the hypothesis that an increased ratio of Firmicutes to Bacteroidetes may make a significant contribution to the pathology of obesity, some recent studies have found controversial results including our previous study for BMI^27,28^.

Interestingly, we also found that pathways related to lysine and glycogen biosynthesis were highly upregulated in the MH group compared with the MU group. In a previous study^29^, lysine level was decreased in nascent metabolic syndrome patients and was negatively correlated with inflammatory biomarkers and cardio-metabolic parameters. Solini et al. found a significant reduction in intracellular glycogen content in hypertensive T2DM patients compared with normotensive T2DM patients^30^. The researchers demonstrated that the reduction in glycogen content in skeletal muscle cells was mainly attributable to impairment of the enzymatic activity of glycogen synthase^30^. Activity of this enzyme was reduced by 35–50% in skeletal muscle cells from T2DM patients compared to those from control subjects^31^. However, because we investigated only the 16S rRNA gene rather than the entire genomes of sampled bacteria, we were only able to infer microbial functions.

Adipose tissue (AT) expansion has been suggested to be a possible determinant of MHO versus MUO status^32^. AT expansion can be mediated by hypertrophy, hyperplasia, or both during obesity. AT hyperplasia could be the preferred expansion mechanism of fat tissue in MHO individuals compared to MUO individuals. Molecular mechanisms controlling hyperplasia and hypertrophy have not been fully elucidated, though microbiota and the gut barrier may regulate AT expansion^32^. Gut microbiota are active and potent modulators of metabolism. Therefore, researchers have suggested that a switch from the MUO to MHO metabolic state may result from gut microbiota remodeling^33^. Additionally, the diversity and richness of microbiota are lower in obese versus lean individuals^34^. In the current study, the characteristics of microbiota in the MH group were more similar to those observed in lean individuals than to those in the MU group. Given our findings, the gut microbiota, due to their association with metabolic health status in obesity, may play a vital role in regulating host glucose homeostasis and lipid metabolism that results in maintenance or remodeling of the gut microbiome and prevention of metabolic abnormalities in the obese population.

There are several limitations to this study. First, we investigated the associations between gut microbiota and metabolic health status in obese and overweight individuals at a single time point. A previous study showed that MHO is an unstable condition for many individuals due to longitudinal changes^35^. In an earlier study, almost half of MHO subjects developed MetS during follow-up^5^. Nevertheless, our results may explain individual heterogeneity in diseases associated with obesity. Second, the study population included only individuals drawn from the Korean population. Therefore, our results may not be generalizable to other ethnic populations with different comorbidities and life-styles. Third, our study was based on 16S rRNA gene sequencing, which provides limited information about bacterial genes and their functions. In addition to collecting descriptive data based on 16S rRNA gene sequencing, it is also crucial to analyze bacterial gene or protein expressions as well as metabolite production to generate hypothesis-driven approaches with clear functional targets. Nevertheless, to the best of our knowledge, this is the first study to evaluate differences in the microbiome according to metabolic health status in obese and overweight Korean individuals.

## Conclusion

In conclusion, there were significant differences in microbial diversity and composition of gut microbiota in metabolically healthy obese individuals compared to metabolically unhealthy obese individuals. Further studies are needed to determine the mechanisms underlying the effects of the microbiome on metabolism in obese individuals, as these mechanisms likely play a key role in preventing metabolic disease.

## Methods

### Study subjects

Participants were recruited from the Kangbuk Samsung Health Study, which is a cohort study of Korean men and women who undergo comprehensive annual or biennial examinations at Kangbuk Samsung Hospital Healthcare Screening Center in South Korea^36^. Fecal samples were collected from 1463 participants aged 23 to 78 years who underwent a comprehensive health examination between June 2014 and September 2014 and who provided informed consent to participate in this study. Participants who met any of the exclusion criteria described below were not included in the analysis (Supplementary Fig. S1). We excluded 716 subjects based on the following criteria: missing data (n = 18); BMI < 23 (n = 616); use of antibiotics within 6 weeks prior to enrollment (n = 55); use of probiotics within 4 weeks prior to enrollment (n = 19); history of cardiovascular disease (n = 24); history of malignancy (n = 52); and samples with less than 2000 sequences (n = 19). Some individuals met more than one exclusion criterion, and a total of 747 participants were included in the final analysis.

The present study was conducted according to a protocol approved by the Institutional Review Board of Kangbuk Samsung Hospital (2013-01-245-12). Written informed consent was obtained from all participants after the nature and possible consequences of the study were explained. All applicable institutional and governmental regulations concerning ethical use of human volunteers were followed during this research. The research was carried out in accordance with the Declaration of Helsinki.

### Data collection and group definitions

Data on medical history, medication use, and health-related behaviors were collected through a self-administered questionnaire. Physical and ultrasound (US) measurements were performed by trained staff during health examinations, and biochemical parameters were measured using whole blood collected during health examinations.

We classified overweight and obese individuals into two groups: a metabolically healthy group (MH) and a metabolically unhealthy group (MU). Overweight and obese were defined according to Asian-specific criteria; overweight as a BMI of 23.0 to 24.9 kg/m^2^ and obese as a BMI of 30 kg/m^2^ or higher^37^. To define metabolic abnormalities, we used the National Cholesterol Education Program Adult Treatment Panel III (NECP-ATP III)^38^. The components for lack of metabolic health required for a subject to be considered MUO were (1) fasting serum glucose ≥ 100 mg/dL or current use of blood glucose lowering agents; (2) blood pressure ≥ 130/85 mmHg or current use of blood pressure lowering agents; (3) hypertriglyceridemia as TG ≥ 150 mg/dL; (4) low HDL-C (< 40 mg/dl in men or < 50 mg/dl in women); or (5) waist circumference > 102 cm in men or > 88 cm in women. Metabolically healthy (MH) status was defined as presence of none of the metabolic abnormalities described above and metabolically unhealthy (MU) status was defined as presence of at least one of the metabolic abnormalities above.

### DNA extraction from fecal samples and 16S rRNA gene sequencing

Fecal samples were immediately frozen at − 20 °C after defecation and stored at − 70 °C within 24 h. DNA extraction from fecal samples was performed within 1 month of storage using the MOBio PowerSoil DNA Isolation Kit (MO BIO Laboratories, Carlsbad, CA, USA) according to the manufacturer’s instructions. Amplification and sequencing were performed to analyze bacterial communities as described previously^39^. Genomic DNA was amplified using fusion primers targeting the variable V3 and V4 regions of the 16S rRNA gene with indexing barcodes. Samples were pooled for sequencing on the Illumina Miseq platform (Illumina, San Diego, CA, USA) according to the manufacturer’s instructions^40^. The DADA2^41^ plugin of the QIIME2 package (version 2019.7, https://qiime2.org)^42^ was used to perform sequence quality control, such as filtering low quality sequences and chimeras, and to construct a feature table of amplicon sequence variants (ASVs). ASVs were generated by denoising with DADA2 and regarded as 100% operational taxonomic units (OTUs). For taxonomic structure analysis, taxonomy was assigned to ASVs using a pre-trained naïve Bayes classifier and the q2-feature-classifier plugin against the Greengene 99% OTUs (version 13_8) of the 16S rRNA sequence database in the QIIME2 package. Contingency-based filtering was used to filter features from a table contingent on the number of samples in which they were observed. We filtered features that were present in only one sample based on the suspicion that these did not represent real biological diversity but were PCR or sequencing errors such as PCR chimeras.

### Statistical analysis

Basic statistical analyses were performed using SPSS version 20.0.0 for Windows (IBM Corp.). For diversity analysis, the feature table was rarefied to 2019 sequences per sample by random subsampling in QIIME2 (Supplementary Fig. S2). To evaluate alpha diversity, we computed the number of ASVs observed in each sample, Shannon index accounting for both evenness and richness, Pielou’s evenness, and Faith’s phylogenetic diversity (PD)^43^. The Kruskal–Wallis test was used as a non-parametric statistical test to test pairwise differences. To measure beta diversity, we used the UniFrac distance^44^ to estimate dissimilarity among group members by incorporating the phylogenetic distances between ASVs. Unweighted and weighted UniFrac distances were calculated to determine presence/absence and abundance of ASVs, respectively. Non-phylogenetic indices such as Bray–Curtis dissimilarities^45^ were also used for abundance data. Pairwise permutational multivariate analysis of variance (PERMANOVA) with 999 random permutations was used to test the significance of differences between groups. Plots of microbial diversity were depicted using ggplot2 package (version 3.3.2) in the RStudio (version 1.3.1073, Boston, MA, USA).

Generalized linear models implemented in multivariate association with linear models (MaAsLin)^46^ of Galaxy of the Huttenhower lab (https://huttenhower.sph.harvard.edu/galaxy/) were used to analyze the association between metabolic abnormalities and gut microbiota. MaAsLin is a multivariate statistical framework that identifies associations between clinical metadata and microbial community abundance. After adjusting for age, sex, and BMI, we compared the abundance of taxa between MHO and MUO. All analyses in MaAslin were performed using default options. Resulting p-values were corrected for multiple comparisons at each phylogenetic level and each personality trait using Benjamini–Hochberg correction (FDR). A q value less than 0.05 was considered statistically significant. Linear discriminant analysis (LDA) effect size (LEfSe) analysis was used to detect potential MH- and MU-specific bacterial markers using LEfSe of Galaxy of the Huttenhower lab^47^.

For functional inferences of the microbial community, we conducted Phylogenetic Investigation of Communities by Reconstruction of Unobserved States 2 (PICRUSt2) (v2.2.0-b)^48^ with ASVs according to the instructions published at https://github.com/picrust/picrust2/wiki. Phylogenetic placement in PICRUSt2 is based on the following three steps: hidden Markov models (HMMER) (www.hmmer.org) to place ASVs, then an evolutionary placement algorithm-NG (EPA-NG)^49^ to determine the best position of these placed ASVs in a reference phylogeny, and genesis applications for phylogenetic placement analysis (GAPPA)^50^ to output a tree of the most likely ASV placements. This results in a phylogenetic tree that contains both reference genomes and environmentally sampled organisms and that is used to predict individual gene family copy numbers for each ASV. PICRUSt2 predictions were supported by Enzyme Classification numbers (EC numbers, as of 21 Jan 2016). We generated PICRUSt2 EC gene family predictions and Metabolic Pathway Database (Metacyc) pathway abundance predictions^51^. Results were visualized in statistical analysis of taxonomic and functional profiles (STAMP) version 2.1.3^52^ and tested using Welch’s *t* test for two groups, MHO vs. MUO. All predictions were corrected for multiple testing (Benjamini–Hochberg method, FDR *q* < 0.05).

## Supplementary information

Supplementary Information.

## Data Availability

The datasets used and/or analyzed during the current study are available from the corresponding author on reasonable request, and the 16S rRNA sequence data are available at the public repository, Clinical and Omics data archives (CODA) in the Korea National Institute of Health by accession number R000635 (https://coda.nih.go.kr/coda/coda/search/omics/genome/selectSearchOmicsGenomePop/R000635.do).
