# Supplementary material for: Gut microbiota and metabolic health among overweight and obese individuals
Source: Sci Rep. 2020 Nov 10;10:19417. doi: 10.1038/s41598-020-76474-8 (PMC7655835; doi:10.1038/s41598-020-76474-8)

# Supplementary Information Files

## Gut microbiota and metabolic health among overweight and obese individuals

Mi-Hyun Kim[1], Kyung Eun Yun[1], Jimin Kim[1], Eunkyo Park[2], Yoosoo Chang[1, 3, 4], Seungho Ryu[1, 3, 4], Hyung-Lae Kim[2], and Han-Na Kim[1, 4, 5*]

[1] Center for Cohort Studies, Total Healthcare Center, Kangbuk Samsung Hospital, Sungkyunkwan University School of Medicine, Seoul, Republic of Korea

[2] Department of Biochemistry, College of Medicine, Ewha Womans University, Seoul, Republic of Korea

[3] Department of Occupational and Environmental Medicine, Kangbuk Samsung Hospital, Sungkyunkwan University School of Medicine, Seoul, Republic of Korea

[4] Department of Clinical Research Design & Evaluation, SAIHST, Sungkyunkwan University, Seoul, Republic of Korea

[5] Medical Research Institute, Kangbuk Samsung Hospital, Sungkyunkwan University School of Medicine, Seoul, Republic of Korea

**Supplementary Figure S1. Enrollment of subjects.** †Some subjects met several exclusion criteria. MH, metabolically healthy; MU, metabolically unhealthy.

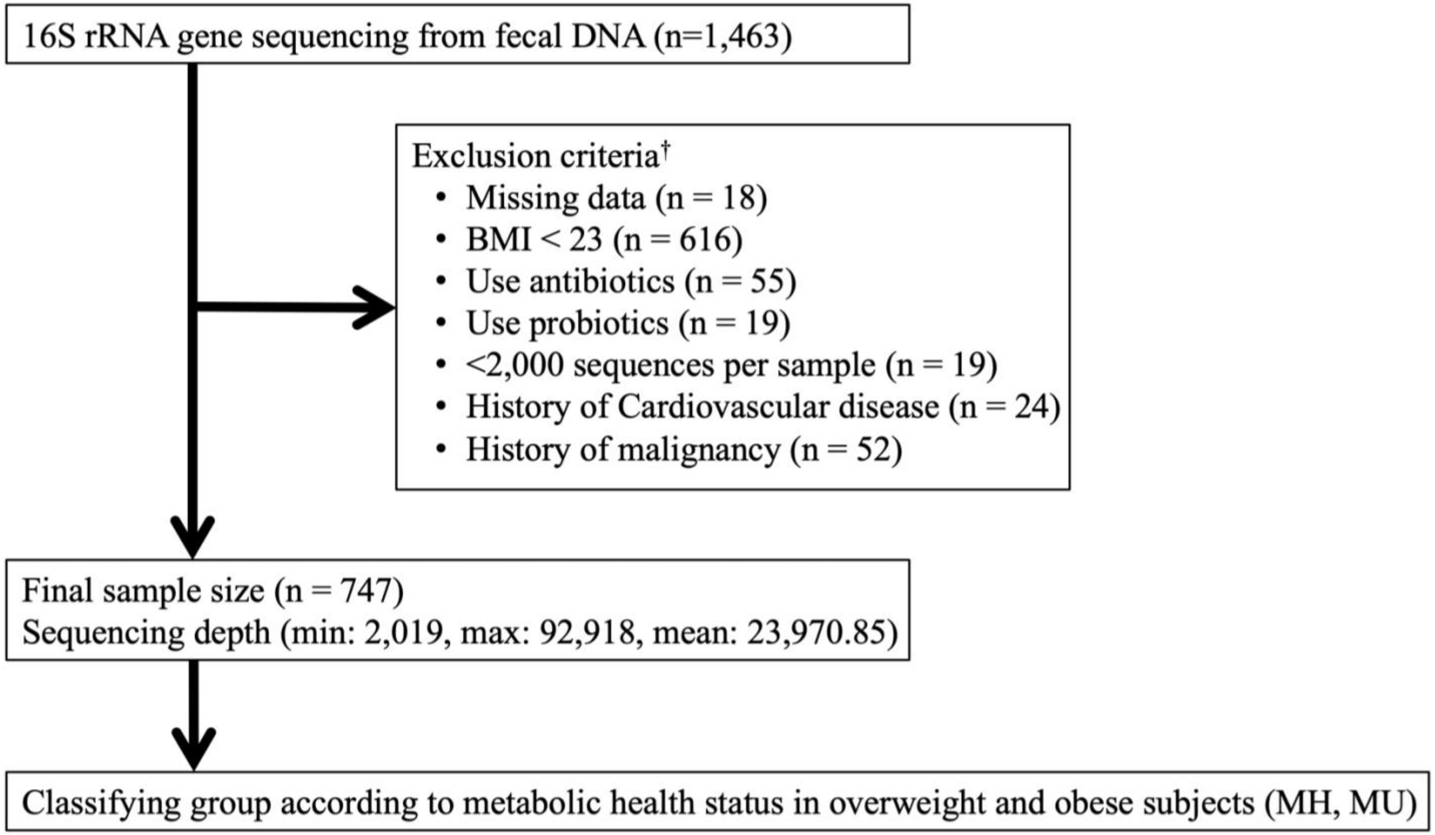

**Supplementary Table S1.** Nutrient characteristics of study participants according to metabolic health in obesity and obese individuals.

| | Total (N = 561) | Metabolically healthy (N = 236) | Metabolically unhealthy (N = 325) | *p*-value |
|---|---|---|---|---|
| Total energy, kcal/day | 1472.8 ± 652.7 | 1522.1 ± 608.7 | 1436.9 ± 681.6 | 0.13 |
| Total protein, g/day | 50.8 ± 25.5 | 51.6 ± 23.9 | 50.1 ± 26.6 | 0.50 |
| Total fat, g/day | 29.2 ± 18.8 | 30.9 ± 19.0 | 28.0 ± 18.7 | 0.08 |
| Total carbohydrate, g/day | 247.7 ± 113.4 | 255.6 ± 105.7 | 241.9 ± 118.5 | 0.16 |
| Total calcium, mg/day | 314.2 ± 195.4 | 314.3 ± 175.3 | 314.2 ± 209.0 | 1.00 |
| Total phosphorus, mg/day | 738.2 ± 344.0 | 745.1 ± 315.4 | 733.1 ± 363.8 | 0.68 |
| Total Vitamin A, ug/day | 341.5 ± 248.2 | 338.4 ± 223.9 | 343.8 ± 264.9 | 0.80 |
| Total sodium, mg/day | 1698.4 ± 1034.4 | 1715.7 ± 948.3 | 1685.8 ± 1093.9 | 0.74 |
| Vitamin B1, mg/day | 0.9 ± 0.4 | 0.9 ± 0.4 | 0.8 ± 0.5 | 0.26 |
| Vitamin C, mg/day | 71.1 ± 58.2 | 73.2 ± 59.7 | 69.5 ± 57.1 | 0.45 |
| Folate, mg/day | 155.0 ± 101.9 | 158.2 ± 99.2 | 152.7 ± 103.9 | 0.53 |
| Retinol, ug/day | 71.9 ± 58.3 | 73.5 ± 53.8 | 70.8 ± 61.5 | 0.58 |
| Fiber, g/day | 3.8 ± 2.3 | 3.8 ± 2.1 | 3.8 ± 2.4 | 1.00 |
| Cholesterol, mg/day | 175.0 ± 138.0 | 180.1 ± 137.9 | 171.3 ± 138.2 | 0.46 |

Mean (standard deviation) and p-value from t-test are shown

**Supplementary Fig. S2. Rarefaction curves based on alpha diversity metrics.** a) The number of observed ASVs, b) Shannon's diversity, and c) Faith's PD, indicated that 2019 sequences per sample are sufficient for capturing the alpha diversity of microbial communities in both MH and MU groups. The x-axis shows the number of sequences per sample. The rarefaction curves construction (10 replicates/depth) were performed using the "diversity alpha-rarefaction" plugin QIIME2.

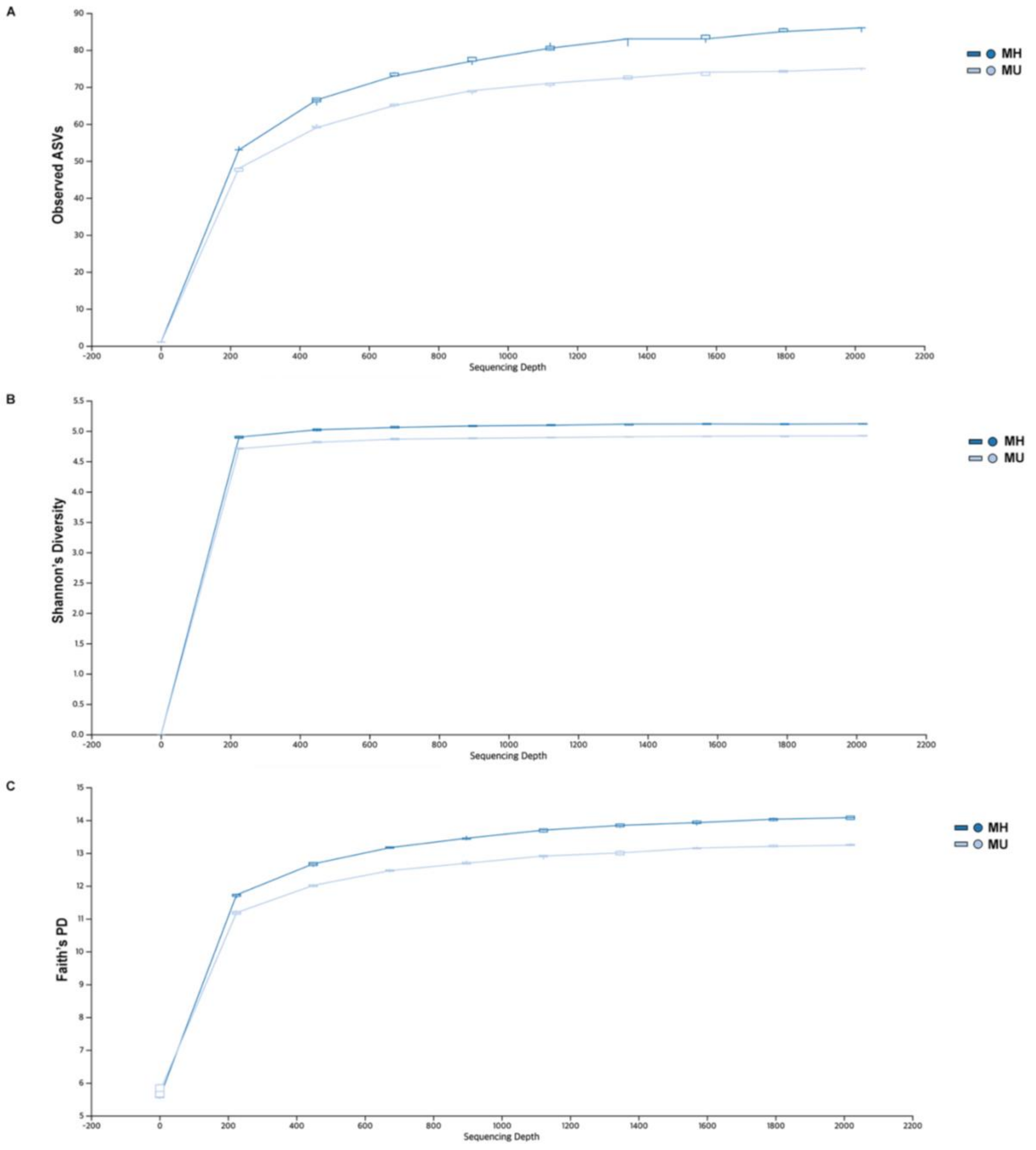

**Supplementary Table S2.** Statistical significance among metabolically healthy non-obese (MHN), metabolically healthy obese (MH), and metabolically unhealthy obese (MU) groups using distance matrices for alpha-diversity.

| | **MHN vs. MH** | **MHN vs. MU** | **MH vs. MU** |
|---|---|---|---|
| **Diversity index** | *p*-value [a] | *p*-value [a] | *p*-value [a] |
| **Observed ASVs** | 0.700 | 0.036* | 0.016* |
| **Faith's PD** | 0.683 | 0.003** | 0.001* |
| **Shannon index** | 0.580 | 0.011* | 0.004* |
| **Pielou's evenness** | 0.886 | 0.495 | 0.442 |

[a] Statistics were calculated using the Kruskal-Wallis Test. * $p < 0.05$, ** $p < 0.01$

**Supplementary Table S3.** Statistical significance among metabolically healthy non-obese (MHN), metabolically healthy (MH), and metabolically unhealthy (MU) groups using distance matrices for beta-diversity.

| | **MHN vs MH** | | **MHN vs MU** | | **MH vs MU** | |
|---|---|---|---|---|---|---|
| **Diversity index** | *Pseudo-F* [a] | *p*-value [a] | *Pseudo-F* [a] | *p*-value [a] | *Pseudo-F* [a] | *p*-value [a] |
| **Bray-Curtis dissimilarity** | 2.137 | 0.001* | 3.834 | 0.001* | 1.565 | 0.007* |
| **Jaccard distance** | 1.499 | 0.001* | 2.777 | 0.001* | 1.509 | 0.001* |
| **Weighted UniFrac distance** | 4.825 | 0.001* | 10.382 | 0.001* | 2.159 | 0.085 |
| **Unweighted UniFrac distance** | 2.677 | 0.003* | 7.250 | 0.001* | 3.294 | 0.001* |

[a] Statistics were calculated using pairwise PERMANOVA with 999 permutations.

* $p < 0.05$, ** $p < 0.01$

**Supplementary Fig. S3. Alpha diversity between males and females.** Diversity was significant for observed features (ASVs) ($p$ = 0.080, Mann-Whitney U-test), phylogenetic diversity ($p$ = 0.013, Mann-Whitney U-test), Shannon index ($p$ = 0.035, Mann-Whitney U-test), and Pielou's evenness ($p$ = 0.486, Mann-Whitney U-test). * $p < 0.05$. Notched boxes indicate interquartile range (IQR) for 25th to 75th percentiles. The median value is shown as a line within the box, and the notch indicates the 95% confidence interval of the median. Whiskers extend to the most extreme value within 1.5 × IQR. Possible outliers are shown as dots.

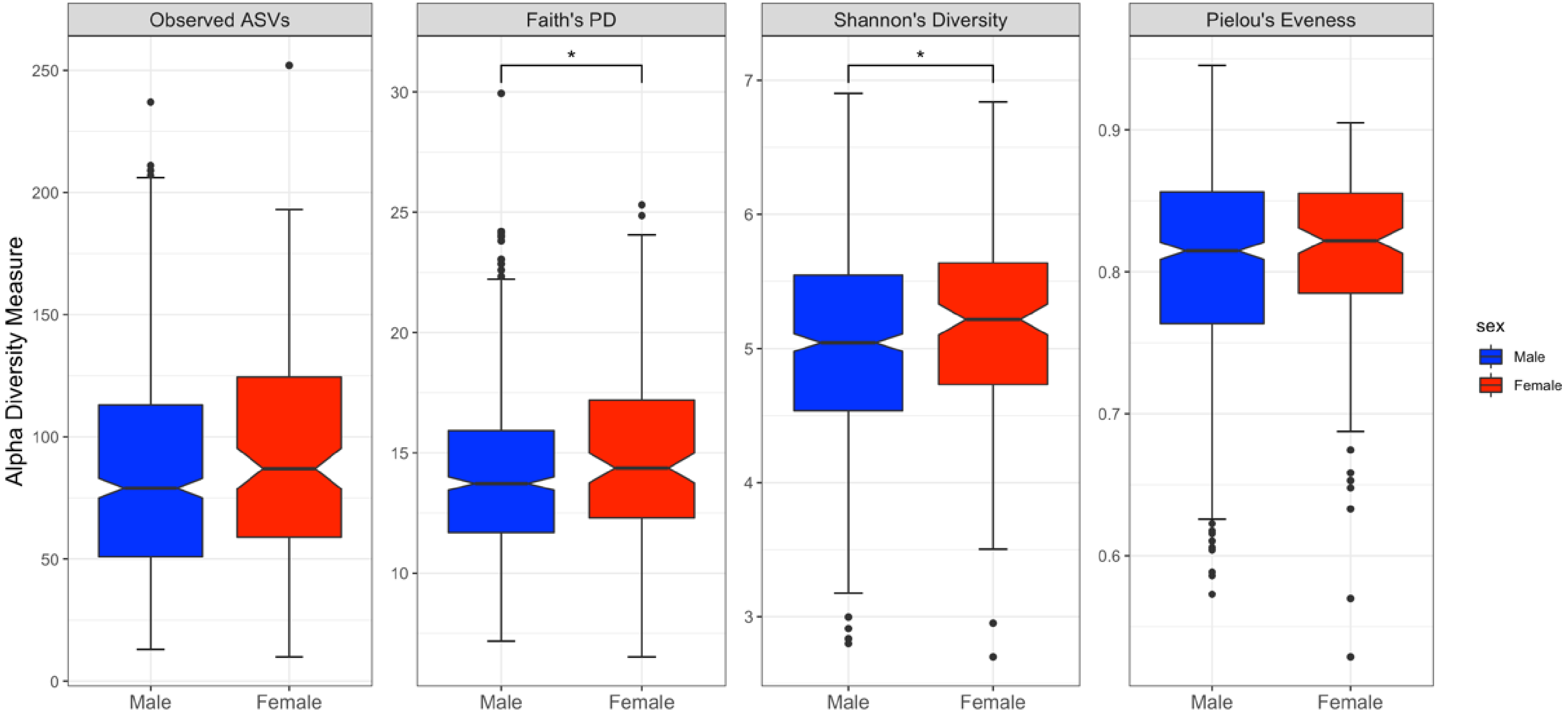

**Supplementary Table S4.** Summary of results for the sex-separated analysis: Alpha diversity

| | Male (mean rank) | | | Female (mean rank) | | |
|---|---|---|---|---|---|---|
| Diversity index | MH | MU | *p*-value [a] | MH | MU | *p* -value [a] |
| Observed ASVs | 315.33 | 282.83 | 0.022* | 86.00 | 71.75 | 0.050* |
| Faith's PD | 319.56 | 279.76 | 0.005** | 88.72 | 69.62 | 0.009** |
| Shannon index | 314.66 | 283.32 | 0.028* | 90.07 | 68.56 | 0.003** |
| Pielou's evenness | 296.24 | 296.69 | 0.975 | 84.18 | 73.17 | 0.130 |

*N* = male, 592; female, 155

MH, metabolically healthy group; MU, metabolically unhealthy group

[a] Statistics were calculated using Kruskal-Wallis Test. * $p < 0.05$, ** $p < 0.01$

**Supplementary Table S5.** Summary of results for the sex-separated analysis: Beta diversity

| | Male (MH vs. MU) | | Female (MH vs. MU) | |
|---|---|---|---|---|
| **Diversity index** | *Pseudo-F* [a] | *p*-value [a] | *Pseudo-F* [a] | *p*-value [a] |
| **Bray-Curtis dissimilarity** | 1.363 | 0.034* | 0.967 | 0.543 |
| **Jaccard distance** | 1.558 | 0.001* | 1.089 | 0.157 |
| **Weighted UniFrac distance** | 1.017 | 0.345 | 2.251 | 0.072 |
| **Unweighted UniFrac distance** | 2.978 | 0.002* | 1.856 | 0.029* |

MH, metabolically healthy group; MU, metabolically unhealthy group

[a] Statistics were calculated using pairwise PERMANOVA with 999 permutations. * $p < 0.05$, ** $p < 0.01$

**Supplementary Table S6.** Association between covariates and the phylum Actinobacteria and their sub-taxa.

| | age | | | sex | | | BMI | | |
|---|---|---|---|---|---|---|---|---|---|
| Taxa | Coef. [a] | *p-value* | *q-value* | Coef. [a] | *p*-value | *q*-value | Coef. [a] | *p*-value | *q*-value |
| p__Actinobacteria | -0.001 | 1.86.E-06 | 2.60.E-05 | -0.036 | 7.53.E-09 | 2.11.E-07 | -0.002 | 9.41.E-02 | 2.03.E-01 |
| p__Actinobacteria;c__Actinobacteria | -0.001 | 8.37.E-07 | 4.02.E-05 | -0.027 | 2.66.E-06 | 6.38.E-05 | -0.002 | 5.87.E-02 | 1.76.E-01 |
| p__Actinobacteria;c__Actinobacteria;o__Bifidobacteriales | -0.001 | 7.37.E-07 | 4.13.E-05 | -0.027 | 2.50.E-06 | 6.99.E-05 | -0.002 | 6.23.E-02 | 1.94.E-01 |
| p__Actinobacteria;c__Coriobacteriia;o__Coriobacteriales | 0.000 | 2.30.E-01 | 4.29.E-01 | -0.001 | 3.78.E-01 | 5.35.E-01 | 0.000 | 7.30.E-01 | 8.02.E-01 |
| p__Actinobacteria;c__Actinobacteria;o__Bifidobacteriales;f __*Bifidobacteriaceae* | -0.001 | 7.37.E-07 | 9.72.E-05 | -0.027 | 2.50.E-06 | 1.65.E-04 | -0.002 | 6.23.E-02 | 1.61.E-01 |
| p__Actinobacteria;c__Coriobacteriia;o__Coriobacteriales;f_ _*Coriobacteriaceae* | 0.000 | 2.30.E-01 | 3.84.E-01 | -0.001 | 3.78.E-01 | 5.25.E-01 | 0.000 | 7.30.E-01 | 8.03.E-01 |

[a] The coefficients from the generalized linear model using MaAsLin.

**Supplementary Fig. S4. Firmicutes/Bacteroidetes ratios in the metabolically healthy (MH) and metabolically unhealthy (MU) groups.**

The *p*-value was calculated using t-test.

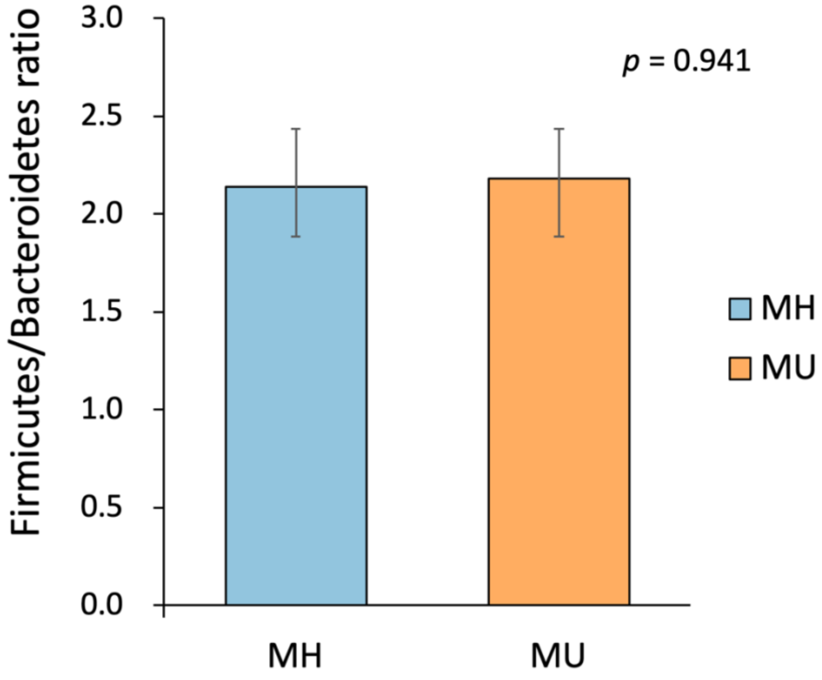

Supplement: Supplementary file 1 — Supplementary Information. [file 41598_2020_76474_MOESM1_ESM.pdf]
